# Medical Properties of the Cotton-Plant

**Published:** 1840-08

**Authors:** 


MEDICINAL PROPERTIES OF THE COTTON PLANT.

We are not aware that the cotton plant, (*Gossypium herbaceum,*) so extensively cultivated in the South for its economical uses, has found a place in the Materia Medica, and should, therefore, probably have remained ignorant of its claims to such distinction, had not our attention been drawn to the subject by a letter from E. F. Bouchelle, M. D., of Columbus, Miss. to Prof. Short. According to the observations of Dr. Bouchelle, the cotton plant displays a particular affinity for the female sexual organs, and is capable of modifying their functions in a remarkable manner. He has exhibited it as a remedy in amenorrhea with decided benefit, five cases of which are mentioned by him, but in terms too general to be satisfactory. He seems to regard it as a specific for this form of catamenial derangement, since he expressly states that it is applicable to every case without regard to the state of the system.

But it is as an oxytocic that the article is most celebrated by Dr. Bouchelle, since in his hands it has proved itself to be nowise inferior to the secale cornutum. He has administered it in several cases of labor, when his hopes of a favorable issue were gloomy indeed, and in every instance he was forcibly impressed with its benignantly specific influence upon the parturient action of the uterus. He has given it *experimentally*, also, in cases of natural labor, and with equally satisfactory results. In such cases, he will allow us to suggest, the remedy maintained a strict neutrality, else it could scarcely have failed to do mischief. In natural labor, the expulsive contractions are justly proportioned, both as to intensity and frequency of recurrence, to the obstacle to be overcome, and they cannot be accelerated or strengthened without risk of injury to both mother and child. Dr. Bouchelle assures us that the cotton plant not only possesses the property of invigorating feeble contrac. tions of the uterine fibres, but that it *originates* expulsive contraction at any period of gestation, and will induce *immediate* abortion when taken in the proper quantity. Should future trials confirm this observation, the gossypium will be entitled to a higher rank as an oxytocic than the secale cornutum, for, it is questionable whether the latter exercises such sway over uterine contraction; at least, we have no satisfactory evidence that it is capable of exciting abortion in a passive and healthy state of the uterus. Dr. Bouchelle avers, however, that the cotton plant is habitually resorted to by slaves in the South for the criminal purpose of inducing abortion, and that it is a fact long and well known in that region that two-thirds, at least, of the likeliest and youngest female slaves are either sterile or invariably miscarry when they do become pregnant, from which he infers, furthermore, that the use of the article destroys the generative capacity. He mentions as a remarkable fact, (and it strikes us in that light too,) that those who resort to it either with the view of inducing abortion or of destroying the generative capacity, experience no detriment to their health therefrom.

The preparation used by Dr. Bouchelle is a decoction made by boiling four ounce of the inner bark of the root in a quart of water until it is reduced to a pint. Dose, a wine-glassful every twenty or thirty minutes as an oxytocic; he does not specify the dose, &c., in amenorrhœa. H. M.

